# A rapid and simple vacuum infiltration system of germinated seeds for plant genetic transformation

**DOI:** 10.3389/fpls.2026.1993488

**Published:** 2026-10-26

**Authors:** Heru Yang, Jiaxue Yu, Weiyan Wang, Ye Chai, Taicheng Jin, Liping Yang

**Affiliations:** 1 The School of Life Sciences, Jilin Normal University, Siping, China; 2 The Key Laboratory of Jilin Province for Plant Resources Science and Green Production, Siping, China

**Keywords:** CRISPR/Cas9 system, GUS, *RUBY*, soybean, vacuum infiltration of germinated seeds

## Abstract

Plant transformation methods play a crucial role in molecular breeding that enhances crop yield and quality. The genetic transformation of soybean is mainly achieved through *Agrobacterium*-mediated methods. To solve the problem of long cycles in plant transformation, this study established and optimized a rapid and simple transformation system, namely the vacuum infiltration system of germinated seeds, in soybean (*Glycine max* (Linn.) Merr.). The expression of β-glucuronidase (GUS) in soybean was achieved using this system, which did not need sterile conditions. The results of reverse transcription quantitative PCR (RT-qPCR) analysis indicated that *the GUS* gene was stably expressed in T1-generation transgenic plants. Compared with traditional transformation methods, this system is rapid and simple and does not require tissue culture procedures. Moreover, to explore the CRISPR/Cas9 gene editing system in combination with vacuum infiltration of germinated seeds, we used the gene editing vector pHEE-*pds* to knock out *the PDS* gene in tobacco (*Nicotiana tabacum* L.). In addition, the visual *RUBY* reporter gene combined with the vacuum infiltration of the cotyledons was applied to the soybean transformation, which effectively improves the screening of candidate transformants.

## 1 Introduction

Innovative research on crop improvement and molecular breeding is crucial for enhancing crop yield and quality. Stable genetic transformation is an important approach for crop breeding research. Studies show that 85% of genetically modified plants are obtained through *Agrobacterium*-mediated genetic transformation (Wu and Zhao, 2017). The transformation methods mainly include disc transformation, floral dip, and vacuum infiltration (Horsch et al., 1985; Clough and Bent, 1998; Li et al., 2017). The vacuum infiltration transformation was first reported in *Arabidopsis thaliana* (Bechtold et al., 1993). The combination of ultrasound and vacuum infiltration avoided the complex tissue culture process and successfully established an efficient transformation system in kidney bean (*Phaseolus vulgaris* L.) (Liu et al., 2005). Researchers first pierced the seeds with a needle and then combined this with vacuum infiltration of *Agrobacterium*, obtaining herbicide-resistant indica rice (Lin et al., 2009).

Soybean (*Glycine max* (Linn.) Merr.), as an important food and oil crop, can provide high-quality protein (Yamada et al., 2012). Since the first genetically modified soybean was obtained (McCabe et al., 1988), the transformation technology used to obtain transgenic soybeans has gradually matured. Researchers used soybean cotyledons as explants and accomplished transformation of soybeans, with a transformation efficiency of 7.23% (Li et al., 2017). The transgenic soybeans were obtained using soybean cotyledons with partial hypocotyls as explants, with a transformation efficiency of 15% (Pareddy et al., 2020). Studies have shown that vacuum infiltration can effectively improve the expression efficiency of foreign genes (Dattgonde et al., 2019). A transformation system for *Rapid-cycling Brassica rapa* (RCBr) was established using either vacuum infiltration or the floral dip method, which will promote development of basic biology research on *Brassica rapa* (Hu et al., 2019).

In this study, a vacuum infiltration system of germinated seeds was established and optimized for the soybean transformation. The concentration of *Agrobacterium* suspensions and the vacuum infiltration conditions were further optimized. Compared with traditional transformation methods, this system is rapid, simple, and tissue-culture-free. Moreover, we knocked out the *PDS* gene in tobacco using vacuum infiltration of germinated seeds combined with CRISPR/Cas9 gene editing system.

## 2 Materials and methods

### 2.1 Experimental materials

The soybean varieties ‘Williams 82’ and ‘Shen Nong 9’ were provided by the Academy of Agricultural Sciences of Jilin Province, and the pDR5-*RUBY* vector was provided by Dr. Yubing He. The CRISPR/Cas9 gene editing vector pHEE-*pds* was provided by Professor Qiwei Zeng. The pCAMBIA1301-*GUS* vector and GV3101 were preserved in our laboratory.

### 2.2 Preparation of *Agrobacterium* suspensions

*Agrobacterium* with GV3101-pCAMBIA1301-*GUS* or pDR5-*RUBY* vector 1 mL was shaken in 50 mL of lysogeny broth (LB) liquid medium, and 50 µL of kanamycin (Kan) (100 mg/mL), 50 µL of rifampicin (Rif) (50 mg/mL), and 80 µL of acetosyringone (AS) (100 mmol/L) were added to the medium. The culture was carried out by shaking at 28°C and 160 r/min for 18 hours. The bacterial cells were collected by centrifugation and the suspension was prepared. Prepare 100 mL of MMA, 100 mL of ddH2O + 1 mmol MES (1 mol/L) + 1 mmol MgCl2 (1 mol/L) + 80 µL of AS. Add 5 µL of Silwet L-77 to the solution and mix well. Use it as a suspension of *Agrobacterium*. Set the bacterial solution concentrations *to OD_600_* values of 0.8, 1.2, 1.6, and 2.0. The bacterial suspension should be placed in the dark for 3 hours.

### 2.3 Vacuum infiltration for germinated seeds

Fifty germinated seeds per experimental group were used for vacuum infiltration in each experiment, and the biological experiments were independently repeated three times. The vacuum infiltration system did not need sterile conditions. In the experimental process, half of the cotyledons were cut off and wounds were made at the cotyledon nodes with a scalpel. Place these two groups of seeds in Petri dishes, respectively, and pour in *Agrobacterium* suspensions until the seeds are completely submerged. Place the Petri dish into the circulating water multi-purpose vacuum pump and set the vacuum treatment time from 5 to 15 minutes. After vacuum infiltration, wash the germinated seeds three times with sterile water. This treatment can avoid the contamination of *Agrobacterium*. Set the vacuum pressures at -0.06, -0.07, and -0.08 MPa, and place the germinated seeds in a petri dish, incubate it in the dark, and perform *Agrobacterium* co-cultivation for 2–3 days, then transplant to soil and cultivate for 3–4 weeks. The seedlings were used for GUS staining detection and analysis of gene expression. The expression efficiency of the *GUS* gene was obtained as the percentage of plants expressing the *GUS* gene among all plants in the T0 generation using RT-PCR detection.

Vacuum infiltration of tobacco germinated seeds does not require wounding the cotyledons. Soak the seeds in *Agrobacterium* suspensions, and directly perform the vacuum treatment at -0.065, -0.075, and -0.085 MPa and *Agrobacterium* co-cultivation for 2–3 days. Then transplant to soil and cultivate for 3–4 weeks. One hundred germinated seeds per experimental group were used for vacuum infiltration in each optimization experiment, and the biological experiments were independently repeated three times.

### 2.4 GUS chemical tissue staining detection and analysis

The infected soybean leaf and stem were placed in GUS staining solution, vacuum-assisted infiltration was carried out, and the plant materials were then incubated overnight in a 37°C incubator under light-proof conditions. The next day, the plant materials were taken out and placed separately in a clean beaker containing 50 mL of Carnoy’s fixative. They were fixed at room temperature (25°C) for 2 hours. Decolorize the fixed plant materials for 3 hours. Handle the plant tissues carefully during the fixation and decolorization processes to prevent damage to the materials. After decolorization, when all plant tissues were free of chlorophyll spots, the staining materials were placed in 75% ethanol for 24 hours. Eight leaves per experimental group were used for vacuum infiltration in each optimization experiment, and the biological experiments were independently repeated three times (n = 8 for leaves).

### 2.5 RT-qPCR, RT-sqPCR detection, and Northern blotting

The total RNA of *GUS* transgenic soybean was extracted using TRIzol reagent. The extraction was carried out 2–3 times with phenol-chloroform, and ethanol was precipitated overnight. Total RNA was reverse transcribed *in vitro* to synthesize complementary DNA (cDNA), and the expression of the *GUS* gene was detected and analyzed by reverse transcription quantitative PCR (RT-qPCR), reverse transcription semiquantitative PCR (RT-sqPCR) detection, and Northern blotting to identify *GUS* transgenic lines. For the RT-qPCR analyses, *the actin* gene was used as the reference gene. The biological experiments were independently repeated three times (n = 3). For the RT-sqPCR analyses, *the GAPDH* gene was used as the reference gene. The biological experiments were independently repeated three times. The methods were as described previously (Yang et al., 2016, 2022).

For the Northern blotting analysis, total RNA was isolated using TRIzol reagent (Invitrogen) according to the manufacturer’s protocols. For the high-molecular-weight RNA gel blot analysis, 10 µg of total RNA was extracted from the inoculated plants and separated on 1% agarose-formaldehyde gels, transferred to Hybond-N+ membranes, and hybridized with digoxigenin (DIG)-labeled probes following the DIG system user’s guide (Roche). The *GUS* probe primers: F(*GUS*): 5’-ATCGTGGTGATTGATGAAACTG-3’; R(*GUS*): 5’-ATCGCTGATGGTATCGGTGT-3’. The RT-qPCR primers for *RUBY* were F(*RUBY*): 5’-AACAGCATCCTTGAGTCTCTTCG-3’; R(*RUBY*): 5’-TTCTCTTGGAGATCTCGCCTTC-3’. The detection primers for the *PDS* gene were F(*PDS*): 5’-GCTCAGTAAAATGCCCCAAA-3’; R(*PDS*): 5’-CCACCCAAACCTGATATTGC-3’. The single-guide RNA (sgRNA) target sequences of *PDS:* sgRNA1: 5’-GTCAAGATGGTCGCTTGCAAAGG-3’; sgRNA2: 5’-CAGGTGAACGTGCTGTCTATTGG-3’ (The highlighted parts are the protospacer adjacent motif (PAM) sites).

## 3 Results

### 3.1 *Agrobacterium*-mediated vacuum infiltration system for germinated soybean seeds

The germinated seeds of soybean (*Glycine max* (Linn.) Merr.) variety ‘Williams 82’ were used as plant materials. The germinated seeds were cut off a cotyledon with a scalpel, and soaked in *Agrobacterium* suspensions (**Figure 1A**) and vacuum infiltrated by *Agrobacterium* with vector pCAMBIA1301-*GUS*. After vacuum treatment, the seeds were co-cultivated for 2 days (**Figure 1B**), transplanted into soil and cultured for 2–3 weeks (**Figure 1C**). The infiltrated soybean plants at 7 days post inoculation (dpi), 14 dpi, and 21 dpi were selected for GUS staining detection. The results of GUS staining showed that the cotyledons, leaves, roots, and stems of soybeans all had obvious blue spots (**Figures 1D–G**), which indicated *GUS* expression in T0_-_generation plants.

**Figure 1:**
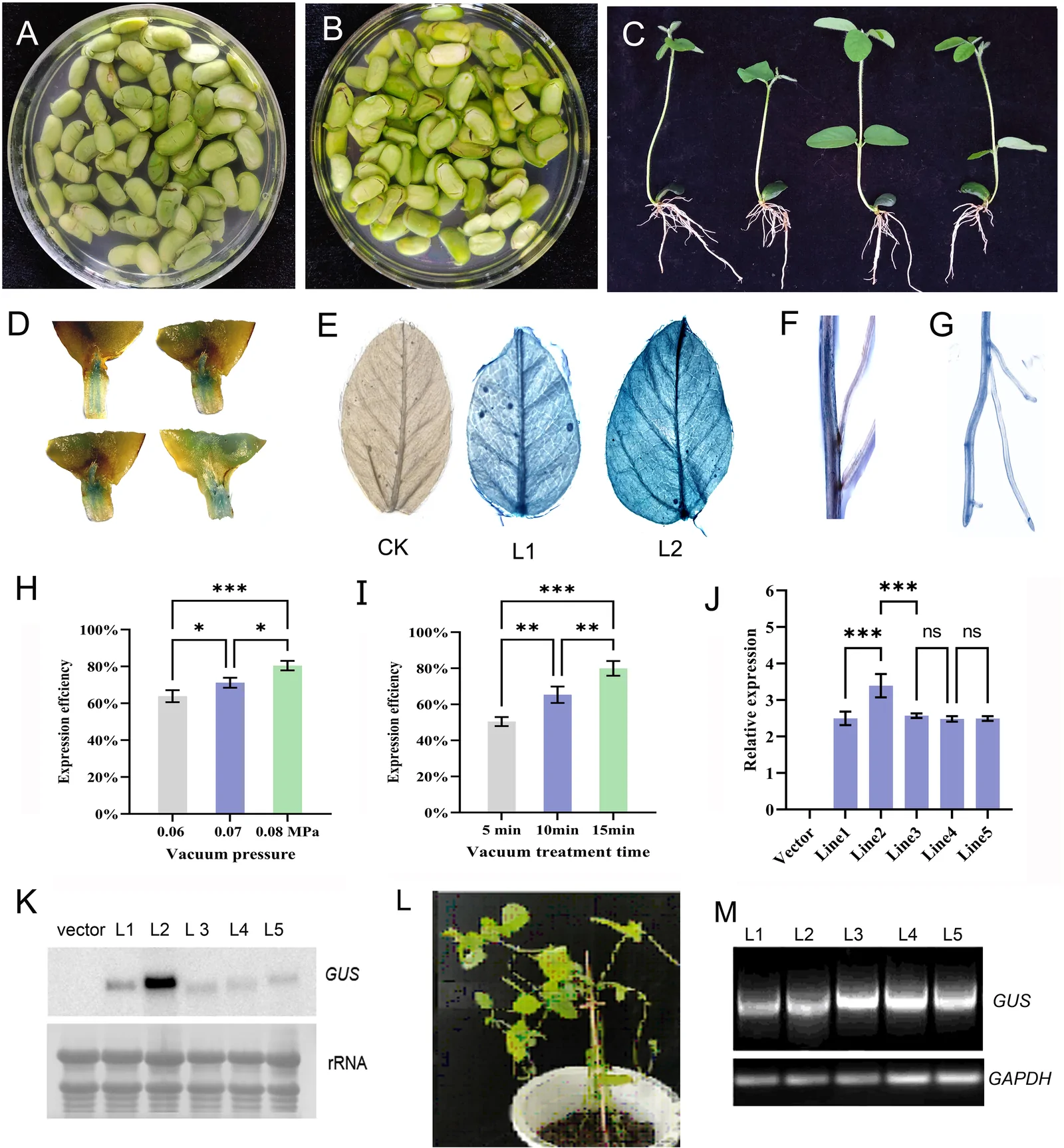
Optimization of vacuum infiltration system of germinated seeds and analysis of *GUS* gene expression. **(A)** The cotyledons of soybeans soaked in *Agrobacterium* suspensions; **(B)** The color of the cotyledons visibly deepened after vacuum infiltration; **(C)** Soybean seedlings were cultivated in soil after vacuum infiltration; **(D)** Detection of *the GUS* gene in cotyledon by GUS chemical tissue staining at 7 dpi; **(E–G)** The *GUS* gene was detected in leaf, root, and stem at 14–21 dpi; **(H)** *GUS* expression efficiency under different vacuum pressures; **(I)** Appropriately increasing the vacuum treatment time improves the expression efficiency; Error bars represent SEs from biological replicates (n = 10 for seeds). **(J)** RT-qPCR analysis of the *GUS* expression in the T1 transgenic lines 1–5; **(K)** Detection of the expression of *GUS* gene in the T1 using Northern blotting; **(L, M)** The transgenic plant of the T2 generation and RT-sqPCR analysis of the expression of *GUS* gene in the T2 transgenic lines 1–5. Error bars represent SEs from three biological replicates (n = 3). *Indicates that the difference is statistically significant at the 5% level (p < 0.05). Asterisks (**) indicate statistically significant differences (two-sample t-test, p < 0.01). Asterisks (***) indicate statistically significant differences (two-sample t-test, p < 0.001).

Studies have shown that an appropriate *Agrobacterium* concentration and vacuum pressure can effectively enhance the expression efficiency of exogenous genes. When the *OD_600_* of *Agrobacterium* was 1.6, the expression efficiency of *the GUS* gene was as high as 82% in the T0 generation. The vacuum pressures were set at -0.06, -0.07, and -0.08 MPa to complete vacuum infiltration on the germinated seed. The results demonstrated that the *GUS* expression efficiency was relatively high when the pressure was at -0.08 MPa (**Figure 1H**), and appropriately increasing the vacuum treatment time effectively improves the expression efficiency (**Figure 1I**). The screened T0 plants were cultivated to harvest the seeds and T1 generations. RT-qPCR (**Figure 1J**) and Northern blotting (**Figure 1K**) of *the GUS* gene were performed in *GUS* transgenic lines. The results further demonstrated expression of *the GUS* gene in T2 soybean plants using RT-sqPCR. The transgenic plants of the T2 generation (**Figure 1L**) and RT-sqPCR analysis of the expression of GUS gene in the T2 transgenic lines 1–5 (**Figure 1M**).

### 3.2 RUBY reporting system combined with vacuum infiltration of soybean cotyledons

The seeds of the soybean variety ‘Shen Nong 9’ were sown in B5 medium (**Figure 2A**). The cut cotyledons were infected with *Agrobacterium* containing the pDR5-*RUBY* vector using the vacuum infiltration method (**Figures 2B, C**). Infiltrated cotyledons were incubated in the dark for 2 days (**Figure 2E, F**). Then, they were transferred to an induction medium with 6-benzylaminopurine (6-BA) and indole-3-acetic acid (IAA) for further cultivation (**Figures 2G, H**), and the cultivation without hormone was used as a control (**Figure 2D**). Germination and rooting occurred simultaneously after induction for 2–3 months (**Figures 2I, J**). The red phenotype of callus tissue gradually appeared, and after the visualization and screening of transformants, the leaves and rhizomes of *RUBY* transgenic plants exhibited a distinct red phenotype (**Figures 2K–M**). RT-PCR further confirmed *RUBY* expression. We found that after both ends of the cotyledon were cut off (**Figure 2C**), the frequency of cotyledon-induced bud and root formation obviously decreased (**Figure 2H**). The experiments to optimize infiltration conditions were conducted, and the results indicated that the expression efficiency of *RUBY* was 25% when the *OD_600_* of *the Agrobacterium* suspension was 0.25. However, when the *OD_600_*of the *Agrobacterium* suspension was 0.45, the contamination rate of *Agrobacterium* obviously increased (**Figure 2N**). When the vacuum pressure was -0.08 MPa, the expression efficiency of *RUBY* was relatively high (**Figure 2O**). The expression of *RUBY* was confirmed by RT-qPCR in T1 plants (**Figure 2P**).

**Figure 2:**
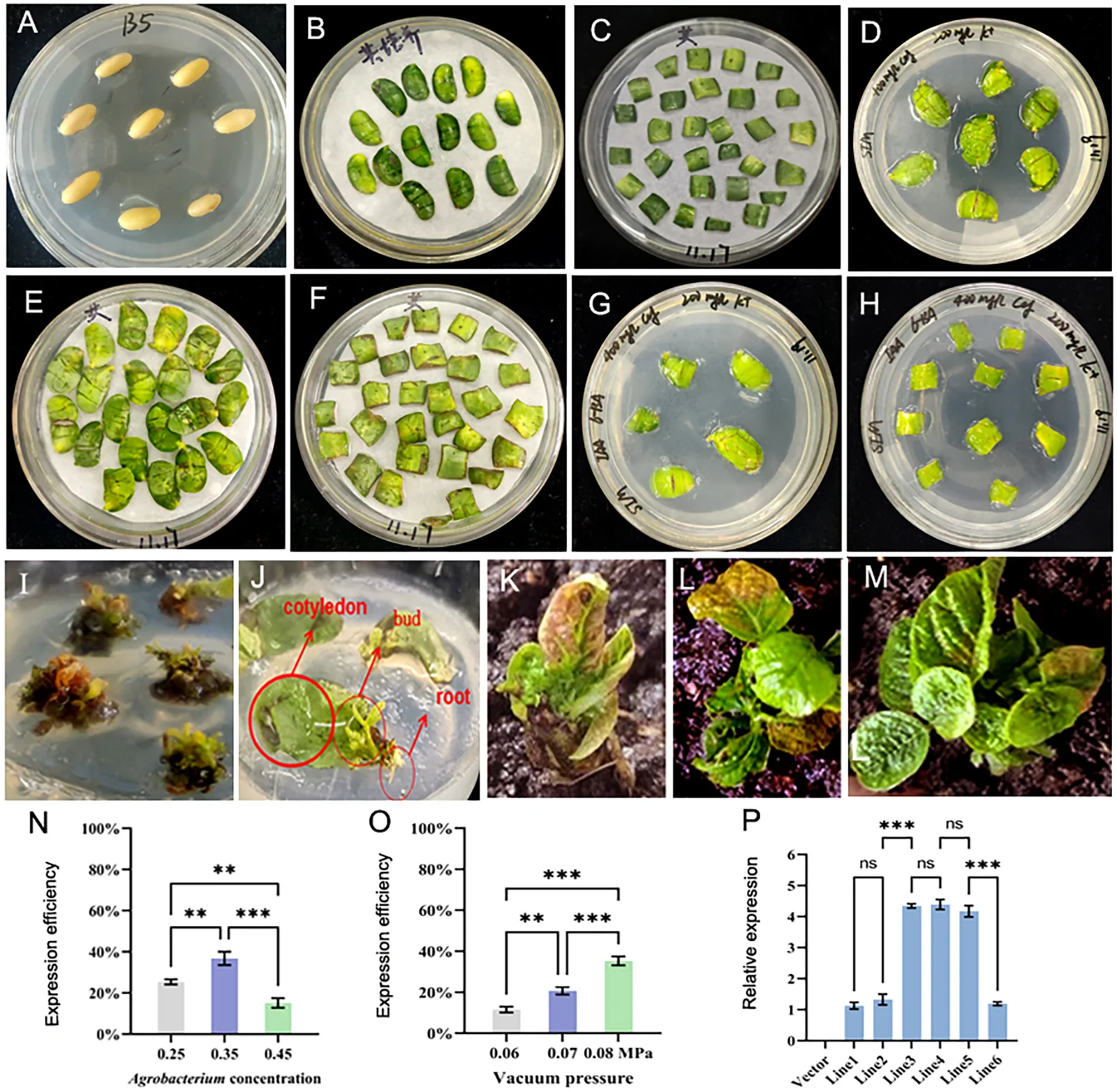
RUBY reporting system combined with vacuum infiltration of soybean cotyledons. **(A)** Sowing in B5 medium; **(B, C)** Cotyledons before co-culture; **(D)** The cotyledons cultivated in hormone-free medium; **(E, F)** Cotyledons after co-culture for 2 days; **(G, H)** Induction culture of soybean cotyledons in medium for 1–2 months; **(I)** The expression of *RUBY* in buds; **(J)** Buds and roots were induced two months later; **(K–M)** The red color phenotype of *RUBY* in soybean seedlings and leaves; **(N)** The expression efficiency of *the RUBY* gene under different *Agrobacterium* resuspension; **(O)** The expression efficiency of *the RUBY* gene under different vacuum pressures; Error bars represent SEs from biological replicates (n = 10 for cotyledons). **(P)** RT-qPCR analysis of the expression levels of *the RUBY* gene in the T1 transgenic lines 1–6. Means identified by different letters are significantly different from each other. Error bars represent SEs from three biological replicates. Asterisks (**) indicate statistically significant differences (two-sample t-test, p < 0.01). Asterisks (***) indicate statistically significant differences (two-sample t-test, p < 0.001).

### 3.3 CRISPR/Cas9 editing system combined with vacuum infiltration of germinated tobacco seeds

To explore CRISPR/Cas9 gene editing system combined with vacuum infiltration of germinated seeds, we used CRISPR/Cas9 gene editing vector pHEE-*pds* to perform knockout of *the PDS* gene in tobacco. Germinated tobacco seeds (**Figure 3A**) were subjected to *Agrobacterium*-mediated vacuum infiltration and co-cultivated with *Agrobacterium* for 2–3 days (**Figure 3B**). A seedling infiltrated with *Agrobacterium* containing an empty vector served as a control (**Figures 3C, D**). The young seedlings had bleached leaves 21 days post-inoculation (dpi) with *Agrobacterium* containing the pHEE-*pds* vector (**Figures 3E–G**). Compared with control plants inoculated with an empty vector (**Figure 3H**), the bleaching phenotype of seedlings was more obvious in 1–2 months after agro-inoculation with pHEE-*pds* vector (**Figures 3I–K**). Mosaic plants were also observed in this study (**Figure 3L**). Further experiments were performed to optimize *Agrobacterium* concentration and vacuum pressure. When *the Agrobacterium* concentration (*OD_600_*) was 1.5, the percentage of bleaching plants in the total number of infiltrated plants reached 35% (**Figure 3M**). The ratio was relatively high when the vacuum pressure was -0.085 MPa (**Figure 3N**). The results of RT-PCR detection showed deletion (641 bp) of *the PDS* gene in plants 9–12, and the expression of *the PDS* gene (1255 bp) in plants 1–8, were used as positive controls (**Figure 3O**). The sequencing analysis further confirmed the detection results of the gene expression.

**Figure 3:**
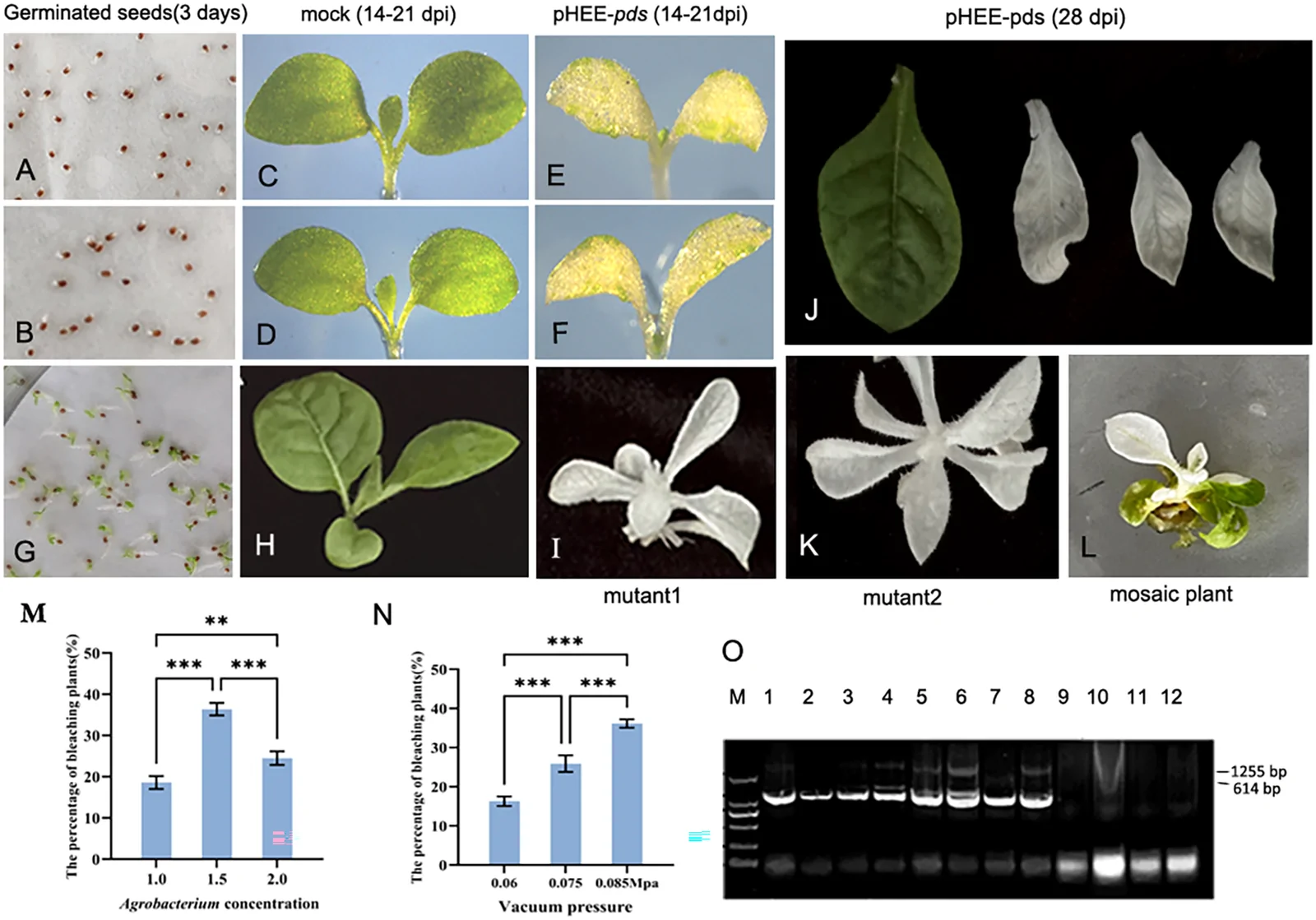
The induction of *pds* mutants using *Agrobacterium-*mediated vacuum infiltration of germinated seeds. **(A)** Germinated tobacco seeds were used for vacuum infiltration; **(B)** *Agrobacterium* co-cultivation for 2–3 days after agro-inoculation; **(C, D)** Seedling cultivation for 14–21 days after inoculation with *Agrobacterium* containing an empty vector, served as a control; **(E–G)** Seedling cultivation for 14–21 days after inoculation with *Agrobacterium* containing the pHEE-*pds* vector; **(H)** Seedling cultivation for 28 days after agro-inoculation with empty vector, served as a control; **(I–L)** Bleaching leaves of *pds* mutant1, mutant2, and mosaic plants for 28 days after agro-inoculation with pHEE-*pds* vector; **(M)** The induction efficiency of *pds* mutants under different *Agrobacterium* concentrations (*OD_600_*); **(N)** The induction efficiency of *pds* mutants under different vacuum pressures; Error bars represent SEs from biological replicates (n = 10 for seeds). **(O)** RT-PCR detection result of the expression of *the PDS* gene in *pds* mutant plants 9–12, plants 1–8 used as the positive controls. Error bars represent SEs from three biological replicates for each experiment. Asterisks (**) indicate statistically significant differences (two-sample t-test, p < 0.01). Asterisks (***) indicate statistically significant differences (two-sample t-test, p < 0.001).

## 4 Discussion

### 4.1 Advantages of the vacuum infiltration system for germinated soybean seeds

The genetic improvement of soybeans is highly dependent on transgenic and genome editing technologies, and an efficient and stable genetic transformation system is an important support. The traditional soybean transformation is restricted by bottlenecks such as strong genotype dependence and low transformation efficiency, making it difficult to be applied on a large scale (Hu et al., 2022). Researchers obtained transgenic soybeans with a transformation efficiency of 9.84% using *Agrobacterium*-mediated tissue culture in combination with gene gun techniques, but the experimental period was as long as seven months (Paes, de. Melo et al., 2020). In this study, the vacuum infiltration system of germinated seeds was adopted to achieve a new transformation system for soybeans. With optimization of *Agrobacterium* concentration and vacuum infiltration conditions, the genetic transformation process required only 1 month. This germinated-seed vacuum infiltration system was faster and simpler than existing methods. From vacuum infiltration of germinated seeds to obtaining T0 plants expressing *GUS*, the genetic transformation process was completed in 3–4 weeks. Moreover, the transient expression efficiency of *the GUS* gene reached 82% in T0 generation, only the gene expression in some seeds from T0 generation could be inherited by offspring. The new system provides a new method for plant genetic transformation and molecular breeding research.

Studies have shown that half a soybean seed with the embryo retained and only the embryo root removed was used as an explant, combined with 10 minutes of ultrasound and 10 minutes of vacuum infiltration, achieving a transformation efficiency as high as 38.0% (Saravanan et al., 2023). Studies have shown that soybean seeds with the germ removed were used as explants, immersed in *Agrobacterium* suspension, and vacuum-treated for 15 minutes. After infiltration, they were transplanted into the soil for cultivation. The *RUBY* gene was expressed in three soybean varieties, without the need for tissue culture and under sterile conditions, with the highest transformation efficiency reaching 9.6% (Cao et al., 2025). Vacuum infiltration can effectively improve the expression efficiency of foreign genes. During the transformation process of oats, with 72 hours of co-culture and 15 minutes of vacuum infiltration, the transformation efficiency reached 21.96% (Dattgonde et al., 2019). In this study, a cotyledon was removed from germinated soybean seeds, and wounds at the cotyledon nodes were used to facilitate *Agrobacterium* infiltration. Reporter gene expression efficiency increased significantly when vacuum infiltration was performed for 15 minutes.

### 4.2 The vacuum infiltration of cotyledons effectively enhances transformation efficiency

Studies have shown that the RUBY reporting system catalyzes the synthesis of betacyanin from endogenous tyrosine in plants with the help of three enzymes, causing the plants to exhibit a red phenotype (He et al., 2020). This system can achieve non-invasive, real-time, and visual tracking of gene expression and genetic transformation processes (Niazian et al., 2023; Chen et al., 2023, 2024; Rowan et al., 2025; Zhu et al., 2025). A simple and rapid quantitative detection method for betacyanin was used in Bens tobacco and corn; the results demonstrated that the content of betacyanin was highly correlated with the expression level of *RUBY* (Pramanik et al., 2024). These studies confirm that the RUBY system has both visual observation and quantitative analysis capabilities, and can be effectively applied to research related to the transient expression and stable transformation of plants.

We employed a visualized *RUBY* reporter gene in combination with a vacuum infiltration method and effectively improved the visualization and screening of candidate transformants. This system of vacuum infiltration of cotyledons required tissue culture and a regeneration period of several months. The vacuum infiltration system of germinated seeds was a tissue-culture-free method, which did not need sterile conditions. Moreover, the experimental period for this system was only one month. Therefore, our results demonstrated that the vacuum infiltration system of germinated seeds was a more conventional and faster regeneration system.

There are a large number of false-positive explants during the process of soybean tissue culture, which is one of the reasons for the low genetic transformation efficiency of soybeans (Yang et al., 2019). Adding 6-benzylaminopurine (6-BA) and gibberellin (GA3) to the co-culture medium can promote the cell division and growth of explants. Adding herbicides to the rooting medium can reduce the emergence rate of non-transgenic roots, thereby improving the transformation efficiency (Cheng et al., 2021). Our study indicates that adding appropriate amounts of 6-BA and IAA to the induction medium can simultaneously increase the germination and rooting rates of soybean cotyledons.

Plant genetic transformation strategies serve as essential tools for the genetic engineering and advanced molecular breeding of plants (Cao et al., 2022). Researchers exploited RUBY-assisted One-shot Tissue-culture-free In-planta Soybean-transformation (ROTIS), which used soybean seedlings as explants, created a precise wound at the growth point, then used vacuum infiltration for *Agrobacterium* infection, and achieved non-invasive tracking throughout the entire growth period (Li Y. et al., 2025). The ROTIS system combines tissue-culture-free with the RUBY system for visual screening. A simple and efficient peanut transformation system was also established using the epicotyls of peanut seedlings at the two-leaf stage as explants, which were immersed in *Agrobacterium* K599 suspension for 30 minutes and then cultured in the dark for 3 days. Transgenic hair-like roots were obtained 30 days later, with a transformation efficiency between 46.2% and 73.7% (Li et al., 2025).

## 5 Conclusions

In this study, a rapid and simple genetic transformation system, namely the vacuum infiltration system of germinated seeds, was established and optimized in soybean and tobacco. Moreover, we knocked out the *PDS* gene in tobacco using vacuum infiltration of germinated seeds in combination with CRISPR/Cas9 gene editing system. This system will provide a new approach for plant breeding and crop variety improvement.

## Data Availability

The datasets presented in this study can be found in online repositories. The names of the repository/repositories and accession number(s) can be found in the article/supplementary material.
